# Influence of Potentially Inappropriate Medication Use on Older Australians’ Admission to Emergency Department Short Stay

**DOI:** 10.3390/geriatrics9010006

**Published:** 2024-01-04

**Authors:** Hoa T. M. Tran, Cristina Roman, Gary Yip, Michael Dooley, Mohammed S. Salahudeen, Biswadev Mitra

**Affiliations:** 1 Department of Pharmacy and Emergency and Trauma Centre, Alfred Hospital, Melbourne, VIC 3004, Australia; c.roman@alfred.org.au; 2 School of Pharmacy and Pharmacology, University of Tasmania, Hobart, TAS 7005, Australia; 3 Department of General Medicine, Alfred Hospital, Melbourne, VIC 3004, Australia; 4 Department of Pharmacy, Alfred Hospital, Melbourne, VIC 3004, Australia; m.dooley@alfred.org.au; 5 Emergency and Trauma Centre, Alfred Hospital, Melbourne, VIC 3004, Australia; biswadev.mitra@monash.edu; 6 School of Public Health & Preventive Medicine, Monash University, Melbourne, VIC 3004, Australia

**Keywords:** polypharmacy, potentially inappropriate medication, emergency, deprescribing, medication review, older population

## Abstract

Older people in the emergency department (ED) often pose complex medical challenges, with a significant prevalence of polypharmacy and potentially inappropriate medicines (PIMs) in Australia. A retrospective analysis of 200 consecutive patients aged over 65 years admitted to the emergency short stay unit (ESSU) aimed to identify polypharmacy (five or more regular medications), assess PIM prevalence, and explore the link between pre-admission PIMs and ESSU admissions. STOPP/START version 2 criteria were used for the PIM assessment, with an expert panel categorizing associated risks. Polypharmacy was observed in 161 patients (80.5%), who were older (mean age 82 versus 76 years) and took more regular medications (median 9 versus 3). One hundred and eighty-five (92.5%) patients had at least one PIM, 81 patients (40.5%) had STOPP PIMs, and 177 patients (88.5%) had START omissions. Polypharmacy significantly correlated with STOPP PIM (OR 4.8; 95%CI: 1.90–12.1), and for each additional medication the adjusted odds of having a STOPP PIM increased by 1.20 (95%CI: 1.11–1.28). Nineteen admissions (9.5%) were attributed to one or more PIMs (total 21 PIMs). Of these PIMs, the expert panel rated eight (38%) as high risk, five (24%) as moderate risk, and eight (38%) as low risk for causing hospital admission. The most common PIMs were benzodiazepines, accounting for 14 cases (73.6%). Older ESSU-admitted patients commonly presented with polypharmacy and PIMs, potentially contributing to their admission.

## 1. Introduction

In 2020/2021, individuals aged 65 years and older accounted for 21% of all emergency department (ED) visits in Australia [1]. Some of these patients were admitted to the emergency short stay unit (ESSU) with the goal of discharge within 24 h [2]. Among ESSU admissions, a substantial portion consisted of older individuals [3] who exhibited polypharmacy [4,5] and were taking potentially inappropriate medications (PIMs) [6].

Within Australia, the prevalence of polypharmacy and PIM usage is notably high and increasing [1,2]. Polypharmacy, defined as the use of more than five medications [7], affects over 40% of individuals aged 50 years or older [8] and around two-thirds of those 75 years or older [9]. Polypharmacy serves as a proxy indicator for inappropriate medication use and has been identified as an independent risk factor for adverse outcomes among ED-presenting patients [10]. It is significantly linked to PIM usage [6] and medication-related problems (MRPs) [11].

PIMs are medications without clear evidence-based indications, that have a higher risk of adverse effects, or that are not cost-effective [12]. Approximately 54.8% of older patients admitted to an Australian tertiary teaching hospital were found to be using multiple PIMs upon admission, with PIMs potentially contributing to hospitalization in 6% of these cases [13]. The prevalence of PIMs varies across different settings, ranging from 22.6% in the community to 43.2% among nursing home residents [14]. A meta-analysis of observational studies revealed a significant association between PIMs and adverse outcomes, including ED visits, functional decline, adverse drug events, hospitalization, and health-related quality of life [14].

Validated tools such as the Medication Appropriateness Index (MAI) [15], Beers criteria [16], and Screening Tool of Older Person’s Prescriptions/Screening Tool to Alert doctors to Right Treatment (STOPP/START) [17] have been employed to identify and improve the appropriateness of medicine prescription in older adults. A systematic review of four randomized controlled trials conducted in four different countries concluded that the application of the STOPP tool reduced the incidence of PIMs, as well as the occurrence of falls, delirium, length of stay, care visits, and costs [18]. However, the utilization of the STOPP/START tool in the ED setting has been limited, with only a few studies documenting its application [19,20]. The ESSU setting offers a distinctive opportunity for a multidisciplinary team to thoroughly assess and manage medication-related concerns, especially for patients who may not require hospital admission, preventing any potential missed opportunities for intervention. Furthermore, the incorporation of partnered medication review and charting by a pharmacist in the emergency short stay creates possibilities for prompt medication assessments by pharmacists. This integration facilitates collaboration with medical staff, enabling the timely identification and management of medication-related issues and reducing medication errors [5,21,22].

The primary objectives of this study were to assess the prevalence of polypharmacy and PIMs using the STOPP/START version 2 [17] tool among older Australians admitted to an ESSU. Furthermore, the study sought to investigate the association between pre-admission PIMs and ESSU admissions.

## 2. Methods

### 2.1. Study Setting

This study was a retrospective observational analysis of 200 patients aged 65 years or older who were admitted overnight to ESSU at Alfred Hospital between October 2021 and December 2021. The Alfred Hospital is a Level 1 trauma center and academic hospital situated in Melbourne, Australia. During the financial year spanning from July 2020 to June 2021, the Alfred ED recorded 64,622 presentations [23], with ESSU managing over 16,000 patients during this period [24]. Patients admitted to the Alfred ESSU typically have hospital stays lasting up to 24 hours, and those who remain overnight are reviewed during morning ward rounds by an ED team consisting of an ED consultant, a junior doctor, and an emergency medicine (EM) pharmacist. The study was approved by the Alfred Hospital Research and Ethics Committee.

### 2.2. Inclusion Criteria

Patients were considered eligible for the study if they were 65 years or older at the time of their admission to the ED. Patient encounters were included if they stayed overnight and underwent medication reconciliation conducted by emergency medicine (EM) pharmacists. All patient encounters meeting these inclusion criteria were included, irrespective of the reason for their care.

### 2.3. Data Sources and Collection

Demographic data were extracted from electronic medical records (Cerner PowerChart) through the Alfred Hospital Data Warehouse. All admissions to the ESSU during the study period for patients aged 65 or older were encompassed. The collected data included age, gender, mode of arrival, triage category, location before admission, length of stay in the ED, and discharge destination.

To further identify eligible patients, a research pharmacist systematically screened cases in reverse chronological order, commencing from December 2021. Subsequently, clinical data for 200 consecutive eligible patients were manually entered into the REDCap database for analysis. This clinical information included allergies, admission diagnoses, pre-existing medical conditions, the number of regular and “as needed” (PRN) medications, as well as any modifications made to medication therapy during the ED stay. The consecutive sampling method was selected for its efficiency and to ensure the inclusion of every eligible patient during the study period.

To detect PIMs, the research pharmacist utilized the validated STOPP/START version 2 criteria [17]. This screening tool encompasses both STOPP (Screen Tool of Older People’s Prescriptions and START (Screening Tool to Alert to Right Treatment (START) [17]. These criteria were applied to the patient’s pre-admission medication lists, which had been previously compiled by an EM pharmacist. The numbers and classifications of STOPP and START PIMs were recorded.

Patients identified by the research pharmacist as potentially having a PIM-related admission underwent an evaluation by an independent expert panel. This expert panel included an emergency physician, a general medicine physician, and a senior EM pharmacist. Their evaluation involved using a two-variable risk assessment matrix [25], which considered both the likelihood and consequences to collectively determine the associated risk levels. The risk levels were predefined as low, moderate, high, and extreme. Consensus was deemed to be reached when all panel members unanimously agreed on the assigned risk levels.

### 2.4. Sample Size

The estimated proportion of patients with PIMs was set at 30%, based on the prevalence range of PIMs between 22.6% and 43.2% [14]. To detect a minimum clinically significant proportion of at least 10% as the threshold for clinical significance, a sample size of 200 was calculated using 90% statistical power and a 95% confidence interval.

### 2.5. Data Analysis

Descriptive statistics were employed to provide a summary of the data. To evaluate the difference between means, the Student’s *t*-test was used, while the Wilcoxon rank-sum test was applied to assess the difference between medians. Differences in proportions were assessed using the chi-square test, or the Fisher’s exact test in cases where the value was less than 5.

The relationship between polypharmacy and STOPP PIMs was analyzed using logistic regression, and the results were presented as odds ratios with 95% confidence intervals (95%CI). These associations were adjusted for potential differences in baseline characteristics (Table 1), except for the number of medications. The adjusted odds ratios (aORs) with 95%CI were reported.

A significant level of *p* < 0.05 was established to indicate statistical significance. All statistical analyses were conducted using Stata v 15.1, College Station, TX, USA.

## 3. Results

### 3.1. Demographics

**Figure 1:**
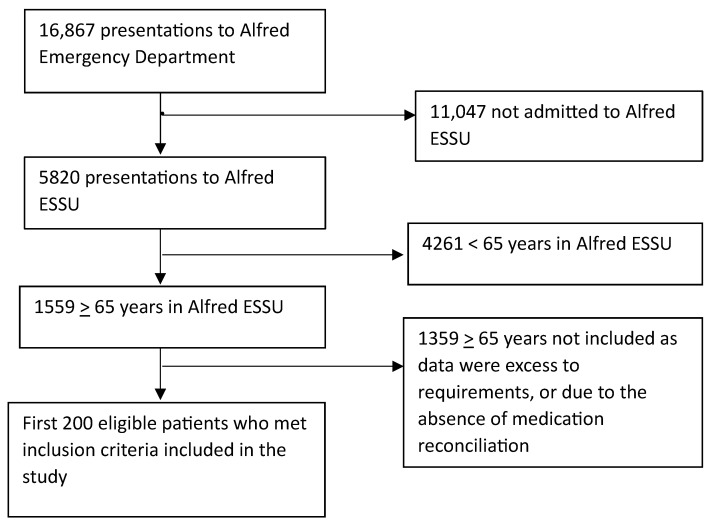
Flowchart of inclusion of patients in the study. ED = Emergency, ESSU = Emergency Short Stay Unit.

From October to December 2021, the Alfred Emergency Department recorded a total of 16,867 presentations. Out of these, 5820 patients presented to ESSU, with 1559 of them being aged 65 years or older. For the study, the first 200 consecutive eligible patients, selected in reverse chronological order, were included (Figure 1).

The mean age was 80.9 years, with females representing 60.5%. More than half (57%) of the study cohort was triaged as ‘urgent’ (category 3). They either came from home (86.5%) or from aged care facilities (13.5%). Various reasons prompted visits to the ED; however, the most common complaint was a fall (34%), followed by pain (17%), and dizziness or syncope (7%). Co-morbidities included hypertension (72%), dyslipidemia (48%), diabetes (29%), arthritis (28%), and depression/anxiety (27%). The breakdown of these baseline characteristics in patients, both with and without polypharmacy, can be found in Table 1.

### 3.2. Potentially Inappropriate Medications Identified Using STOPP/START Criteria

A total of 185 patients, which corresponds to 92.5% of the study population, were found to have at least one PIM. The details of the distribution of each STOPP and START criterion can be found in Table 2.

Among the participants, 161 (80.5%) individuals were identified as having polypharmacy. In the polypharmacy group, the mean age was 82 years, which was significantly higher than the mean age of 76 years observed in the non-polypharmacy group (*p* < 0.001). Additionally, the median number of regular medications for those in the polypharmacy group was nine, compared to three for those in the non-polypharmacy group (*p* < 0.001).

The STOPP criteria were detected in 81 (40.5%) patients. Among these patients, a total of 131 STOPP PIMs were identified from the pre-admission medication list, as detailed in Table A1. The most frequently encountered STOPP PIMs included benzodiazepines (15.5%), medications prescribed without an evidence-based clinical indication (11%)—such as low-dose aspirin without a history of ischaemic heart disease or stroke, and pantoprazole without a history of gastroesophageal reflux or peptic ulcer disease— and duplicate medications (5.5%) such as concurrent oxazepam and temazepam or concurrent meloxicam and ibuprofen.

Polypharmacy was significantly associated with STOPP PIMs, with an odds ratio (OR) of 4.8 (95%CI: 1.90–12.1, *p* = 0.001). After adjusting for the patient’s age, this association remained statistically significant, with an adjusted odds ratio (aOR) of 4.51 (95%CI: 1.75–11.56, *p* = 0.002). Furthermore, for each additional medication, the adjusted odds of experiencing PIM were increased by a factor of 1.20 (aOR 1.2, 95%CI: 1.11–1.28).

A total of 177 patients (88.5%) were identified as having START omissions. The cumulative count of START omissions amounted to 384 (Table A2). Notably, polypharmacy was not found to be significantly associated with START omissions (OR 2.11; 95%CI: 0.80–5.61, *p* = 0.13). Among the START criteria, the most prevalent omission was related to pneumococcal vaccination (71%), followed by seasonal influenza vaccination (42%), and vitamin D/calcium supplementation in patients with a history of osteoporosis/fractures (29.5%).

The length of stay in ED was not found to be associated with the presence of STOPP PIM, as indicated by a mean difference of 0.04 days (95%CI: −0.06 to 0.09). Similarly, the length of stay in ED was not associated with START omissions, with a mean difference of 0.01 days (95%CI −0.06 to 0.09).

### 3.3. Potential ESSU Admissions Related to PIMs

Nineteen admissions (9.5%) to ESSU were determined by an expert panel to be associated with 21 PIMs (Table A3). Notably, all these cases were linked to STOPP PIMs, with benzodiazepines being implicated in 14 (73.6%) of these admissions. The expert panel categorized eight of these PIMs (38%) as high risk, five (24%) as moderate risk, and eight (38%) as low risk in terms of their potential impact.

## 4. Discussion

The study revealed a significant prevalence of polypharmacy and PIMs within the older ESSU patient cohort, which may have potentially contributed to ED admissions. These findings emphasize the critical importance of implementing multidisciplinary approaches to detect and address PIMs, particularly among patients with polypharmacy, to reduce the risk of hospitalization.

The proportion of older patients with polypharmacy in this study was comparable to a study conducted in Spain, which reported a rate of 93.8% [27]. However, it was significantly higher than the findings of an Italian ED study, where only 30.3% of patients were taking six to nine prescription medicines [4]. This variation could potentially be attributed to the high consumption of complementary medicines by Australians [8]. Notably, patients with polypharmacy in this study had a median of nine regular medications, aligning with results from other studies involving hospitalized patients [5,28].

Our study’s overall finding of 92.5% of patients with at least one STOPP/START PIM aligns with similar findings in recent studies [27,29] that also employed STOPP/START criteria. These studies reported that 97% of patients had at least one PIM upon discharge from an Australian hospital [29], and 81.5% of Spanish hospitalized patients had at least one PIM [27].

In our study, 40.5% of patients were found to have at least one STOPP PIM, a proportion similar to the findings of a systematic review conducted by Thomas et al. [28], which reported a weighted average rate of 42.8% with at least one PIM in community patients and 51.8% in hospitalized patients. However, our study’s results were comparatively lower than those of other similar studies employing the STOPP v2 criteria. Specifically, these studies reported a prevalence of 62.5% upon discharge [29] and 51% in hospitalized individuals aged 85 years and over in an Australian ED setting [30].

The most frequently identified STOPP PIM in our study was benzodiazepines, accounting for 15.5% of cases. This finding was in line with the results from Thomas et al.’s [28] study, which reported a weighted average of 19% in community patients. However, our study observed a lower prevalence compared to the 31.8% found in Bare et al.’s study [27]. Considering that benzodiazepine usage is linked to an elevated risk of falls [31], a complaint presented by 34% of the study participants, it is crucial to identify and establish the causal relationship between inappropriate benzodiazepine use and falls. Dementia and/or delirium were comorbidities present in 16% of the study population. Long-term use of benzodiazepines has a detrimental effect on cognition and should be avoided or gradually discontinued [32]. Proton pump inhibitors (PPIs) were commonly identified as PIMs in other studies [27,28,30]; however, these were not prevalent in our study. This discrepancy is likely due to a lack of information or incomplete documentation regarding the indication and duration of PPIs in the electronic medical records.

In our study, a high proportion of patients (88.5%) had at least one START omission, which was similar to findings in other studies that also included vaccine omissions [29,30]. However, it is important to note that the high rates of START omissions in our study may be due to a lack of documentation or availability of immunization status in My Health Record or electronic medical records, potentially not accurately reflecting omission rates. Excluding vaccine omissions, this proportion would be 44.5%, which is consistent with results from other studies, ranging between 36.6% [28,29] and 44.6% [30].

The omission of vitamin D and calcium supplements in patients with a history of osteoporosis or fractures was next most common after vaccine omissions. This finding aligns with results from other studies [27,30]. While there are potential benefits to patients, osteoporosis prevention and health promotion activities are not frequently undertaken in ED. This could be attributed to competing demands on clinicians, resulting in a lack of prioritization for these activities [33].

Our study observed an association between polypharmacy and STOPP PIMs, which is consistent with findings from previous studies [34,35,36,37]. The adjusted relative risk (RR) between the total number of medications per patient and STOPP v2 criteria PIM was found to be 1.06 (1.05–1.07, *p* < 0.001) in one of these studies [35]. Lau et al. [36], who used partial Beers criteria, also documented an increased odds ratio (OR) between the number of medications used and PIMs, with an OR of 6.39 for five to six medications, increasing to 18.43 for nine or more medications. Similarly, Bao et al. [37], who also employed the Beers list, found an OR of 2.23 for patients taking 8–10 medications and 6.19 for those taking 15 or more medications.

Almost 1 in 10 (9.5%) admissions to ESSU in our study may have been related to PIMs, a proportion in line with findings from other studies [38,39]. The majority (63%) of these PIM-related admissions in our study were attributed to benzodiazepines and falls, which aligns with results from Eshetie et al. [40], where 55.5% of PIM-related admissions were linked to falls associated with the use of falls risk medications in aged care residents. Furthermore, our expert panel assessed that 38% of these PIMs posed a high risk of leading to hospital-related admissions.

The analysis did not include an examination of the number of PIMs at discharge, as it fell beyond the project’s scope. The STOPP/START criteria version 2 was employed in this study; however, since then, the new STOPP/START criteria version 3 [41] has been published. This updated version encompasses 190 criteria, a notable increase from the 114 criteria in version 2. The revisions are reflective of the growing evidence base in pharmacotherapy and therapeutic options for older people. Consequently, it is recommended that future studies adopt the updated criteria for a more comprehensive evaluation.

The findings of this study underscore the importance of focusing on the identification, prevention, and management of PIMs to reduce the risk of hospitalization. Deprescribing PIMs in the ED presents several challenges, including time constraints and limited information and follow-up resources [42,43,44]. To address these challenges and enhance patient care, various strategies can be employed, including those aimed at preventing admission and addressing needs during the admission process:

1. Comprehensive medication reviews by EM pharmacists: EM pharmacists can play a pivotal role in conducting thorough medication reviews, identifying PIMs, and initiating deprescribing interventions [45,46]. Interventions made by EM pharmacists such as medication reconciliation and providing deprescribing suggestions to at-risk patients have led to a ten-fold increase in deprescribing of PIMs by primary care physicians [47].
2. Education on deprescribing: Providing education to healthcare professionals, including pharmacists and doctors, is essential to empower them with the knowledge and skills needed for effective deprescribing [43,44]. Education interventions provided to patients and their caregivers may lead to a reduction in medication use and need to be targeted to prevent admission to ED [48,49].
3. Computer-based decision support (CDS) tools: Computer-based decision support tools have been demonstrated to enhance prescribing practice for older individuals in the ED. These improve recommended dose administrations, promote deprescribing of PIMs, and reduce the incidence of inappropriate prescriptions [50].
4. Inclusion of geriatricians in ED: Involving geriatricians in ED can provide specialized input in medication decisions for older patients [51].
5. Collaboration and follow-up with community-based providers: Collaborative efforts and effective communication with community-based doctors and pharmacists are crucial to ensure seamless transitions in patient care and medication management [52]
6. Utilization of Home Medication Review (HMR) or Hospital-Initiated Medication Review (HIMR): These services can be valuable in reviewing and optimizing medication use [53]. According to data from the Australian Commission on Safety and Quality in Health Care, only about 5.4% of people aged 75 years and over had at least one government-subsidized service for a Residential Medication Management Review (RMMR) or a Home Medicine Review (HMR) in 2018–19 [54]. There is a pressing need to enhance access to these services and develop strategies to improve the uptake of pharmacist recommendations. The Society of Hospital Pharmacists (SHPA) has also established a pathway for HMR referrals for patients seen in the ED [55].

In addition to the aforementioned strategies for identifying, assessing, and managing PIMs, it is imperative to assess the patient’s capacity to safely manage their medications. Individuals with dementia are at a higher likelihood of experiencing comorbidities, potentially leading to polypharmacy [56]. Cognitive impairment can significantly impact a patient’s ability to plan, organize, and execute tasks related to medicine management, thereby elevating the risk of errors and adverse events [57]. Strategies to enhance medication management in such cases include simplifying medication regimens by either reducing the overall number of prescribed medications or minimizing the frequency of medication administration, employing dose administration aids, and implementing reminders and prompts [56,57].

This study’s primary strengths lie in its unique focus on the ED setting and the utilization of expert panel assessments to evaluate PIM-related admissions. In contrast, many prior studies were conducted in hospital or community settings, often lacking the advantages of expert panel assessments for potential PIM-related admissions. The study does have several limitations, including its single-center nature, the use of non-probable sampling, the collection of data at a single time, and the examination of polypharmacy appropriateness, which was conducted through descriptive analysis.

## 5. Conclusions

Among older patients admitted to an emergency short stay unit (ESSU), both polypharmacy and potentially inappropriate medications (PIMs) were prevalent. Benzodiazepines appeared as the most frequently identified STOPP PIMs and were also strongly associated with hospital admission. The findings underscore the importance of preventing PIMs and prioritizing the identification and management of PIMs within routine medication reviews in the emergency department (ED).

## Figures and Tables

**Table 1:** Baseline characteristics of older ESSU patients (n = 200).

	Presence of Polypharmacy	Absence of Polypharmacy	*p*-Value
	N = 161 (80.5%)	N = 39 (19.5)	
Age, mean (SD), years	82.0 (8.4)	76.2 (9.1)	<0.001
Sex, n (%)			0.19
Male	60 (37.3%)	19 (48.7%)	
Female	101 (62.7%)	20 (51.3%)	
Mode of presentation, n (%)			0.89
Ambulance	119 (73.9%)	30 (76.9%)	
Community transport	9 (5.6%)	1 (2.6%)	
Other	33 (20.5%)	8 (20.5%)	
ATS, n (%)			0.62
1	1 (0.6%)	0 (0%)	
2	16 (9.9%)	6 (15.4%)	
3	90 (55.9%)	24 (61.6%)	
4	52 (32.3%)	9 (23.0%)	
5	2 (1.2%)	0 (0%)	
Accommodation before presentation, n (%)			0.99
Home	139 (86.3%)	34 (87.2%)	
Other	22 (13.7%)	5 (12.8%)	
Co-morbidities, n (%)			
Hypertension	126 (78.3%)	16 (44.4%)	
Dyslipidaemia	89 (55.3%)	6 (16.7%)	
Arthritis	53 (32.9%)	3 (8.3%)	
Diabetes	52 (32.3%)	6 (16.7%)	
Atrial fibrillation/arrhythmia	50 (31.1%)	4 (11.1%)	
Depression/anxiety	49 (30.4%)	6 (16.7%)	
Osteoporosis	47 (29.2%)	4 (11.1%)	
Ischaemic heart disease	46 (28.6%)	2 (5.6%)	
Asthma/COPD	43 (26.7%)	2 (5.6%)	
Cancer	33 (20.5%)	4 (11.1%)	
Allergy present, n (%)			0.48
Yes	72 (44.7%)	15 (38.5%)	
No	89 (55.3%)	24 (61.5%)	
Number of allergies *, median (IQR)	1 (1–3)	2 (1–3)	0.85
Total number of regular home medications, median (IQR)	9 (6–12)	3 (2–4)	<0.001
Total number of PRN medications, median (IQR)	1 (0–2)	0 (0–1)	0.002

ATS (Australian Triage Scale) is a clinical tool used to establish the maximum waiting time for medical assessment and treatment of a patient: ATS 1—immediate, ATS 2—10 min, ATS 3—30 min, ATS 4—60 min, ATS 5—120 min [26]. * If allergy present; COPD = chronic obstructive pulmonary disease; ESSU = emergency short stay nit; PRN = as needed.

**Table 2:** Distribution of each STOPP and START criterion to determine PIM.

Category of STOPP/START	STOPP Proportion of Patients 81 (40.5%)	START Proportion of Patients 177 (88.5%)
A	33 (40.7%)	37 (20.9%)
B	4 (4.9%)	7 (3.9%)
C	6 (7.4%)	10 (5.6%)
D	27 (33.3%)	0
E	2 (2.5%)	35 (19.8%)
F	2 (2.5%)	1 (0.6%)
G	1 (1.2%)	0
H	1 (1.2%)	0
I	1 (1.2%)	87 (49.1%)
L	4 (4.9%)	-
Total	81 (100%)	177 (100%)

STOPP category: A = indication of medication, B = cardiovascular system, C = antiplatelet/anticoagulant drugs, D = central nervous system and psychotropic drugs, E = renal system, F = gastrointestinal system, G = respiratory system, H = musculoskeletal system, I = urogenital system, L = analgesic drugs,. START category: A = cardiovascular system, B = respiratory system, C = central nervous system and eyes, D = gastrointestinal system, E = musculoskeletal system, F = endocrine system, G = urogenital system, H = analgesics, I = vaccines.

## Data Availability

The datasets generated and/or analyzed in this study are available from the corresponding author upon reasonable request and approved by the Alfred Hospital Research and Ethics Committee.

## Appendix A

**Table A1:** Classification of STOPP PIM identified. (n = 200) (n, %).

D5. Benzodiazepines for ≥4 weeks. K1. Benzodiazepines (sedative, may cause reduced sensorium, impair balance).	31 (15.5%)
A1. Any drug prescribed without an evidence-based clinical indication.	22 (11%)
A3. Any duplicate drug class prescription, e.g., two concurrent NSAIDs, SSRIs, loop diuretics, ACE inhibitors, or anticoagulants (optimisation of monotherapy within a single drug class should be observed prior to considering a new agent).	11 (5.5%)
L2. Use of regular (as distinct from PRN) opioids without concomitant laxative (risk of severe constipation).	7 (3.5%)
D1. TriCyclic Antidepressants (TCAs) with dementia, narrow angle glaucoma, cardiac conduction abnormalities, prostatism, or prior history of urinary retention (risk of worsening these conditions).	5 (2.5%)
L3. Long-acting opioids without short-acting opioids for break-through pain (risk of persistence of severe pain)	4 (2%)
A2. Any drug prescribed beyond the recommended duration, where treatment duration is well defined.	3 (1.5%)
D14. First-generation antihistamines (safer, less toxic antihistamines now widely available).	3 (1.5%)
E6. Metformin if eGFR < 30 mL/min/1.73 m^2^ (risk of lactic acidosis).	3 (1.5%)
F2. PPI for uncomplicated peptic ulcer disease or erosive peptic oesophagitis at full therapeutic dosage for >8 weeks (dose reduction or earlier discontinuation indicated).	3 (1.5%)

**Table A2:** Classification of START omission identified. (n = 200) (n,%).

I2. Pneumococcal vaccine at least once after age 65 according to national guidelines.	142 (71%)
I1. Seasonal trivalent influenza vaccine annually.	84 (42%)
E3. Vitamin D and calcium supplement in patients with known osteoporosis and/or previous fragility fracture(s) and/or bone mineral density T-scores more than −2.5 in multiple sites.	59 (29.5%)
E2. Bisphosphonates and vitamin D and calcium in patients taking long-term systemic corticosteroid therapy.	16 (8%)
A3. Antiplatelet therapy (aspirin or clopidogrel or prasugrel or ticagrelor) with a documented history of coronary, cerebral, or peripheral vascular disease.	12 (6%)
A7. Beta-blocker with ischaemic heart disease.	11 (5.5%)
B1. Regular inhaled b2 agonist or antimuscarinic bronchodilator (e.g., ipratropium, tiotropium) for mild to moderate asthma or COPD.	11 (5.5%)
C3. Acetylcholinesterase inhibitor (e.g., donepezil, rivastigmine, galantamine) for mild-moderate Alzheimer’s dementia or Lewy body dementia (rivastigmine).	11 (5.5%)
E4. Bone anti-resorptive or anabolic therapy (e.g., bisphosphonate, strontium ranelate, teriparatide, denosumab) in patients with documented osteoporosis, where no pharmacological or clinical status contraindication exists (bone mineral density T-scores ->	11 (5.5%)
A6. Angiotensin converting enzyme (ACE) inhibitor with systolic heart failure and/or documented coronary artery disease.	9 (4.5%)
E5. Vitamin D supplement in older people who are housebound or experiencing falls or with osteopenia (bone mineral density T-score is >−1.0 but <−2.5 in multiple sites).	8 (4%)
A5. Statin therapy with a documented history of coronary, cerebral, or peripheral vascular disease, unless the patient’s status is end-of-life or age is >85 years.	7 (3.5%)
A8. Appropriate beta-blocker (bisoprolol, nebivolol, metoprolol or carvedilol) with stable systolic heart failure.	6 (3%)
A1. Vitamin K antagonists or direct thrombin inhibitors or factor Xa inhibitors in the presence of chronic atrial fibrillation.	5 (2.5%)

**Table A3:** Details of likely PIM-related ESSU admission as assessed by expert panel.

	STOPP/ START PIM (Y/N)	Risk Rating (Assessed by Expert Panel)
1. Oxybutynin in patient presented with delirium/confusion	Y	Moderate
2. Long-term temazepam in patient presented with confusion	Y	Moderate
3. Long-term oxazepam in patient presented with fall and fracture	Y	High
4. Long-term temazepam in patient presented with fall	Y	High
5. Amitriptyline (no apparent indication) in patient with dementia and presented with epilepsy	Y	Moderate
6. Long-term temazepam in patient presented with fall	Y	Low
7. Long-term clonazepam in patient presented with fall (no history of epilepsy) and benztropine in patient presented with fall	Y	High/Low
8. Long-term oxazepam in patient presented with fall	Y	Low
9. Long-term temazepam in patient presented with fall and fracture	Y	High
10. Long-term oxazepam in patient presented with fall	Y	Low
11. Thiazide diuretic (hydrochlorothiazide) in patient with current significant hyponatraemia	Y	High
12. Long-term temazepam in patient presented with fall and fractures	Y	High
13. Long-term diazepam in patient presented with fall	Y	Low
14. Selective alpha-1 selective alpha blockers in patient with symptomatic orthostatic hypotension	Y	High
15. Long-term oxazepam in patient with fall and fracture	Y	High
16. Long-term oxazepam and diazepam in patient presented with fall	Y	Low
17. Diazepam in patient presented with falls due to alcohol intoxication	Y	Low
18. Indomethacin in patient with acute on chronic kidney disease, and chlorthalidone in patient with acute on chronic kidney disease	Y	Moderate/Moderate
19. Benzodiazepines in acute or chronic respiratory failure pO_2_ < 8.0 kPa (60 mmHg) in patient with SOB/CCF exacerbation	Y	Low

pO_2_ = partial pressure of oxygen.
